# Are hospital environments an underestimated source for Gram-negative infections in critically ill patients? A non-multi-drug-resistant *Klebsiella pneumoniae* outbreak in an Irish intensive care unit

**DOI:** 10.1016/j.infpip.2025.100499

**Published:** 2025-11-21

**Authors:** S. Ali, G. Macori, N. Mullane, B. Jayaseelan, O. Donoghue, S. Fanning, K. Schaffer

**Affiliations:** a Department of Clinical Microbiology, Royal College of Surgeons in Ireland, Dublin, Ireland; b Department of Clinical Microbiology, St Vincent's University Hospital, Dublin, Ireland; c School of Biology and Environmental Science, University College Dublin, Dublin, Ireland; d School of Public Health, Physiotherapy & Sports Science, University College Dublin, Dublin, Ireland

**Keywords:** Handwashing sinks, Healthcare-associated infections, Intensive care unit, *Klebsiella pneumoniae*, *Klebsiella variicola*, Outbreak

## Abstract

*Klebsiella pneumoniae* is a major cause of healthcare-associated infections. Although colonisation with Gram-negative bacteria in hospitalised patients is well recognised, the relative contributions of patient-to-patient versus environment-to-patient transmission remain unclear. Most outbreak investigations focus on multi-drug-resistant (MDR) strains. This study investigates a non-MDR *K. pneumoniae* outbreak in an intensive care unit (ICU) to examine the role of the hospital environment in nosocomial transmission. Clinical isolates of Klebsiella spp. were obtained from six patients between January 2021 and June 2021. Environmental swabs were collected from handwashing sinks in patient and preparation rooms, 3 months apart. Whole-genome sequencing (WGS) assessed singlenucleotide polymorphism (SNP)-based relatedness using literature-informed thresholds. WGS identified five distinct clusters of genetically related K. pneumoniae isolates, linking clinical and environmental sources. The closest relationships (2—5 SNPs) were observed between patient and sink isolates within the same room, consistent with recent transmission or a shared source. Additional clusters (5—23 SNPs) involved isolates from sinks in different rooms, indicating environmental persistence and potential inter-room dissemination. Two *Klebsiella varicola* subspecies variicola bloodstream isolates from spatially distinct patients differed by only 2 SNPs, forming an additional cluster consistent with a common clonal lineage. Following enhanced daily sink disinfection and staff education, no further clinical acquisitions were identified. WGS demonstrated genetic relatedness between nonMDR Klebsiella spp. strains and ICU environmental isolates, underscoring the role of environmental reservoirs in transmitting antimicrobial-susceptible Gram-negative organisms and the importance of targeted surveillance beyond MDR settings.

## Introduction

*Klebsiella pneumoniae*, a member of the Enterobacterales order, is a clinically significant Gram-negative organism (GNO) and a leading cause of healthcare-associated infections (HCAIs), responsible for approximately 10% of HCAIs worldwide [1,2]. Its importance has grown with the global emergence of multi-drug-resistant (MDR) strains, particularly those producing extended-spectrum β-lactamases (ESBLs) and carbapenemases [1]. However, even in the absence of antimicrobial resistance, *K. pneumoniae* possesses numerous features that make it a formidable nosocomial pathogen. It can asymptomatically colonise mucosal surfaces of patients and healthcare workers (HCWs), and crucially, it can persist in the healthcare environment [1]. This combination of colonisation potential and environmental survival facilitates ongoing cross-transmission in clinical settings, even in the absence of overt lapses in infection prevention and control (IPC) practices.

Intensive care units (ICUs) are particularly susceptible to such transmission due to the presence of critically ill patients with multiple risk factors for infection, including breaches in natural barriers due to invasive devices or surgery, immunosuppression and repeated or prolonged exposure to broad-spectrum antimicrobials [3]. The complexity of care in these settings demands robust, multi-faceted IPC strategies, which must extend beyond standard hand hygiene and patient contact precautions.

Environmental contamination is increasingly recognised as a major contributor to bacterial cross-transmission, colonisation and infection [[4], [5], [6]]. Weinstein, in his landmark study, estimated that up to 20% of ICU-acquired infections could be attributed to environmental sources [7,8]. This contamination is not limited to high-touch surfaces and shared medical devices but includes the broader environment, including paper records, linen, ventilation systems and most notably, hospital plumbing and water/wastewater systems. These aquatic environments serve as ideal niches for GNOs, providing the moisture and nutrients needed to support biofilm formation, microbial persistence and horizontal gene transfer [9].

Multiple investigations have implicated hospital drainage systems, particularly sinks, P-traps and wastewater pipes, as long-term reservoirs and sources of HCAIs [10,11]. These systems can harbour numerous GNOs, with pathogens transmitted to patients via splash-back, aerosolisation or indirect contact through contaminated hands and surfaces. Biofilm formation within plumbing makes complete eradication difficult and even extensive infrastructural interventions such as full plumbing replacement have failed to eliminate pathogens like carbapenemase-producing Enterobacterales (CPE), as demonstrated in numerous published outbreak reports [6,[10], [11], [12]].

A striking feature of the current literature, however, is the overrepresentation of MDR organisms (MDROs) in reported sink-related outbreaks [11,13]. This is likely a reflection of surveillance bias; outbreaks caused by MDROs are more readily detected due to routine screening programs and the clinical urgency associated with MDR infections. In contrast, antimicrobial-susceptible or non-MDR strains may go unnoticed, either dismissed or not flagged for further investigation due to the absence of concerning antimicrobial resistance patterns. In several documented cases, non-MDR organisms were traced to water/wastewater systems but were not immediately investigated due to the absence of antimicrobial resistance phenotypes, suggesting that the true burden of environmental transmission is likely underestimated [11]. This oversight is problematic as it ignores the real potential for antimicrobial-susceptible organisms to cause nosocomial outbreaks via environmental transmission routes. Environmental transmission dynamics are not exclusive to MDROs; rather, the hospital water/wastewater system acts as a permissive reservoir for both antimicrobial-susceptible and resistant pathogens alike [5,11].

The coronavirus disease 2019 (COVID-19) pandemic has further complicated this landscape. Despite heightened IPC measures and the use of personal protective equipment (PPE), ICUs globally reported numerous MDRO outbreaks during the pandemic period, highlighting that environmental routes of transmission may bypass standard contact precautions [14]. More broadly, multiple outbreak reports have shown that conventional IPC measures are often insufficient to halt transmission linked to hospital water/wastewater systems. For example, a recent Dutch outbreak of Verona integron-encoded metallo-β-lactamase (VIM) -producing *Pseudomonas aeruginosa* persisted despite intensified cleaning, retraining and engineering modifications and was only controlled following complete removal of contaminated water fixtures [15]. Similar experiences internationally demonstrate that interventions such as enhanced disinfection, ward closures or patient isolation frequently fail to address sink-based reservoirs, with control achieved only through source removal or biofilm-targeted approaches [[16], [17], [18], [19], [20], [21], [22]]. In this context, it becomes even more critical to consider the role of aquatic environmental reservoirs, which are not mitigated by PPE, and to assess both MDR and non-MDR organisms when investigating suspected outbreaks.

In this study, we describe an outbreak of antimicrobial-susceptible *Klebsiella* spp. in a COVID-19 ICU. By integrating whole-genome sequencing (WGS) with environmental sampling, we explore the potential role of sink drains in cross-transmission and aim to better understand the contribution of the hospital environment, particularly water/wastewater systems, to the dissemination of GNOs.

## Methods

### Study setting

The study was undertaken in an ICU in a tertiary care referral centre in Dublin, in the Republic of Ireland. The ICU investigated comprises two distinct wards – ICU-A and ICU-B.

**Figure 1:**
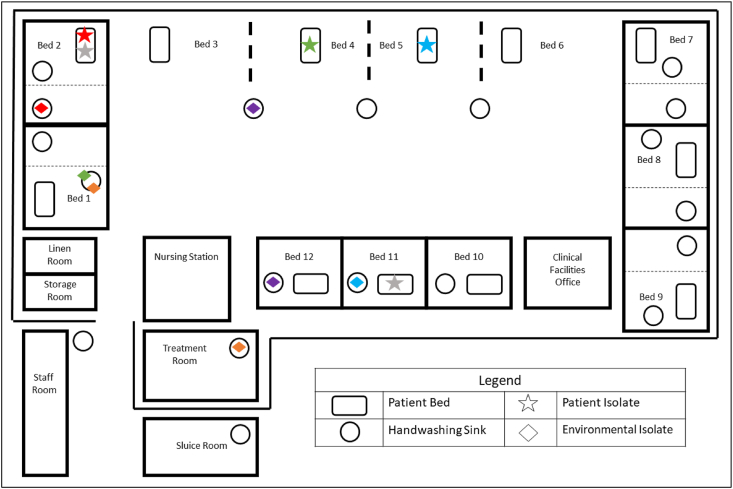
Schematic of ICU-A showing the spatial distribution of Klebsiella isolates studied. ICU: intensive care unit.

ICU-A consists of 17 handwashing sinks and 12 inpatient beds. Five beds are lobbied single rooms, containing handwashing sinks within the room as well as the antechamber and three beds are single rooms with individual handwashing sinks within each room, with the remaining four beds on the open floor with three handwashing sinks interspersed between them. Another handwashing sink is situated in the treatment room where medications are prepared for administration. A schematic of ICU-A is provided as Figure 1 within the Results.

ICU-B consists of eight handwashing sinks and seven inpatient beds. Two beds are lobbied single rooms with handwashing sinks within the room as well as the antechamber, and the remaining five beds are on the open floor with four handwashing sinks interspersed between them.

The two units are separated by a link corridor and are staffed by distinct cohorts of HCWs. However, staff movement between units may occur during critical situations, such as when specialised clinical expertise is required or during periods of significant staff shortages.

### Study design

During 2021, repeated isolation of antimicrobial-susceptible or non-MDR *K. pneumoniae* from blood and/or sputum cultures of different patients who developed infection ≥48 h after COVID-19 ICU admission prompted a combined retrospective and prospective investigation to determine whether the hospital environment contributed to these HCAIs.

An antimicrobial-susceptible or non-MDR strain was defined as an isolate expressing resistance to ≤ two different classes of antimicrobial agent tested and which harboured no common resistance mechanisms – namely AmpC β-lactamases, ESBLs or carbapenemases [23].

Initial outbreak investigations, including assessments of shared equipment, procedural spaces and staffing overlap, revealed no commonalities among affected patients. Comprehensive audits of environmental cleaning and adherence to IPC protocols demonstrated full compliance; no double gloving was observed, and hand hygiene and equipment decontamination compliance was consistently high. This underscored the need for further environmental assessment to identify potential sources of the outbreak. In this regard, this study also served as a quality improvement initiative aimed at identifying potential environmental reservoirs of HCAIs.

### Patient data

Clinical and laboratory data of all patients admitted to the COVID-19 ICU from January 2021 to June 2021 inclusive were reviewed. Patients were included in the study if *Klebsiella* spp. was isolated from blood and/or respiratory specimens as these were deemed clinically significant and consistent with a diagnosis of bloodstream or respiratory tract infection. Infections were diagnosed based on clinical presentation, supported by relevant biochemical and radiological investigations where applicable. Rectal screening cultures for *Klebsiella* spp. were not performed, and urinary isolates were excluded due to the absence of clinically diagnosed urinary tract infections. All patients were catheterised, and catheter-specimen urinary isolates were considered more likely to reflect catheter colonisation. Isolates from other sites were similarly excluded when there was no clear clinical evidence of infection. A pseudonymised data set was collected, including date and site of positive culture, bed allocation, COVID-19 status and clinical outcome, which included either clinical improvement or cure – defined by the sterilisation of clinical cultures and/or resolution of infection-related symptoms – or poor clinical response, recurrence or infection-related mortality.

### Environmental sampling

All handwashing sink drains within the ICU were swabbed on the same day (in line with local IPC policy), 3 months apart throughout 2021. Environmental sampling was performed according to national guidance provided by the Healthcare-Associated Infection and Antimicrobial Resistance (HCAI/AMR) Team [24]. Briefly, a flocked nylon swab was rotated down the sink drain and its tip incubated in 100 mL of diluent (buffered saline) for 24 h at 37 ^o^C. Selective and differential agars were then inoculated with 10 μL of the enriched broth and incubated for an additional 24 h at 37 ^o^C. Bacteria were subsequently identified, and antimicrobial susceptibility testing (AST) was performed to allow phenotypic comparisons. If environmental strains related to the outbreak are detected or high-risk organisms such as CPE were identified, enhanced disinfection protocols are implemented under the guidance of local IPC and clinical microbiology teams. With the consequent isolation of *Klebsiella* spp. from handwashing sinks, WGS analysis was employed to assess genomic relationships among these clinical and environmental isolates.

### Bacterial identification and antimicrobial susceptibility testing

Specimens included blood cultures, sputum samples and environmental surveillance swabs. Bacteria were identified using the bioMérieux VITEK-MS matrix-assisted laser desorption/ionisation-time of flight (MALDI-TOF) instrument [25]. AST was done through VITEK® as per manufacturer's instructions [26]. Minimum inhibitory concentrations were interpreted according to the European Committee on Antimicrobial Susceptibility Testing clinical breakpoints (version 14) [27].

### Whole-genome sequencing

Genomic DNA was purified from overnight cultures in tryptic soy broth (TSB) using the DNeasy® UltraClean® Microbial Kit (Qiagen, Manchester, UK) according to the manufacturer's instructions. The quality and purity of genomic DNA (gDNA) was assessed using a Qubit fluorometer (Thermo Fisher, Newport, Ireland). For library preparation, 20 ng/μL of gDNA from each isolate was used as input for the NEBNext Ultra II FS DNA Library Preparation Kit for Illumina (New England Biolabs, Hitchin, UK), following the manufacturer's protocol for large fragment sizes. Sequencing was performed on the MiSeq platform (Illumina, San Diego, CA, USA) using the V3 reagent kit (2 × 300-bp paired-end reads).

### Genomic analysis

The reads generated were first assessed using FastQC (version 0.11.9) [28] and quality-filtered using FastP (version 0.20.1). De-novo genome assemblies were generated using SPAdes (version 3.12.0). The resultant draft genome sequences were screened for antimicrobial resistance-encoding genes using ABRicate (version 1.0.1) [29] interrogating the Comprehensive Antibiotic Resistance Database [30] and ResFinder [31]. Isolates were further characterised using Kaptive (version 2.0), which was employed to assign multi-locus sequence types (STs) and to identify capsule (*K*) and lipopolysaccharide (*O*) loci [32]. Genome annotation was performed using Prokka (version 1.14.6) [33], and data visualisation was conducted in RStudio [34]. Phylogenetic relationships between isolates were assessed using a core-genome single-nucleotide polymorphism (SNP) approach to ensure high-resolution genetic relatedness analysis. Parsnp (version 1.7.4) was employed for core-genome alignment and SNP calling [35]. A maximum likelihood phylogenetic tree was generated directly within Parsnp, and the tree was visualised using FigTree (version 1.4.5) [36].

### Data availability

This Whole Genome Shotgun project has been deposited at DDBJ/ENA/GenBank under the accession PRJNA1126319.

## Results

The study was initiated in February 2021 when two patients admitted to ICU-A with COVID-19 pneumonitis each developed an ICU-acquired *K. pneumoniae* bacteraemia simultaneously with isolates displaying identical antibiograms. This prompted an epidemiological investigation into potential sources of these isolates.

Twenty *Klebsiella* spp. isolates were examined during the study period – two *Klebsiella variicola* subspecies *variicola* and 18 *K. pneumoniae*. Eight environmental isolates were obtained from six spatially distinct handwashing sinks. Twelve clinical isolates were retrieved from six individual patients. *K. pneumoniae* was cultured from blood from four patients (patient 1, patient 3, patient 4 and patient 5), from sputum from one patient (patient 6) and from both blood and sputum from another (patient 2) (Table I).

**Table I:**
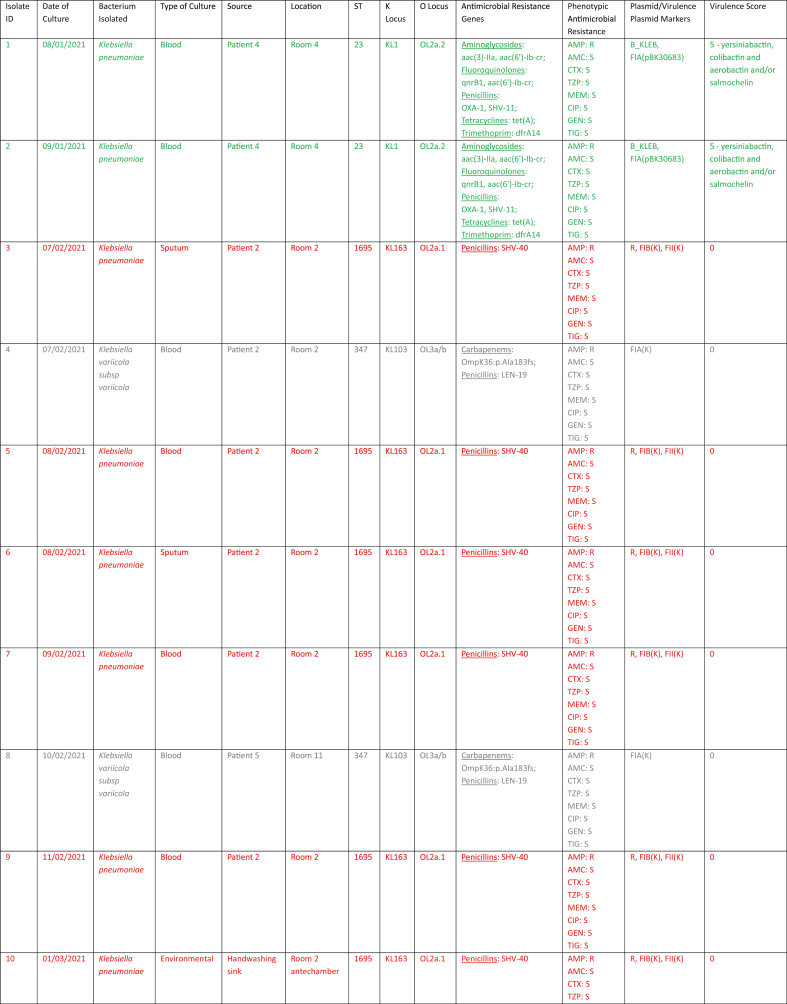

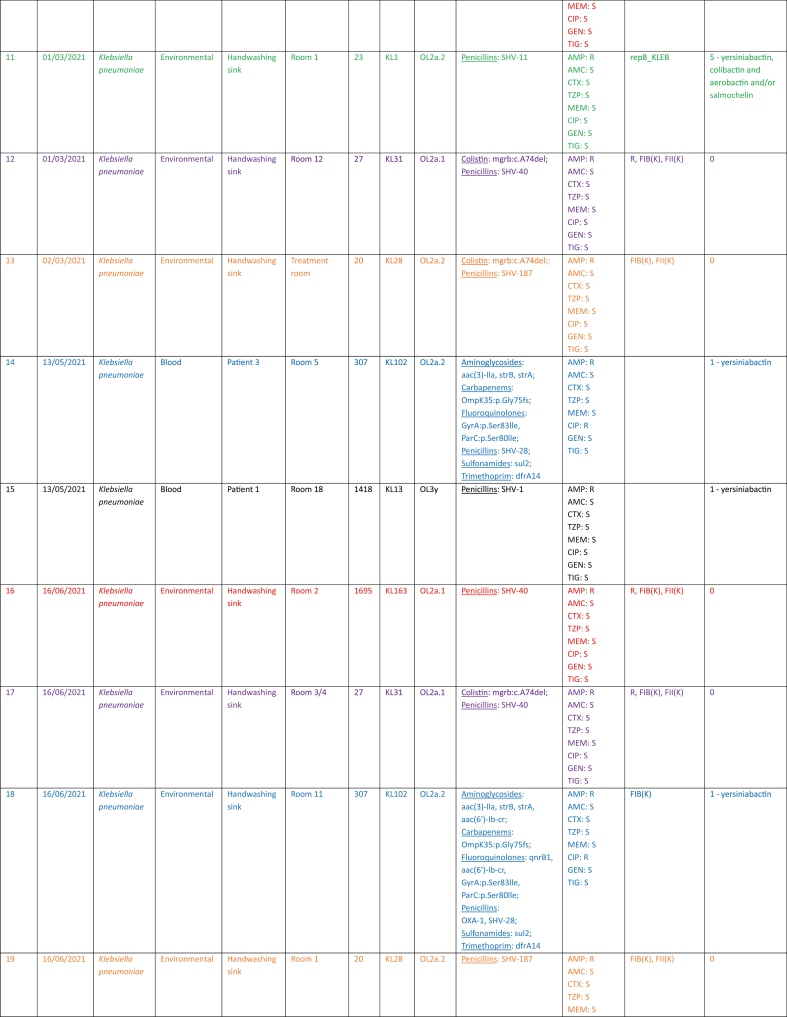

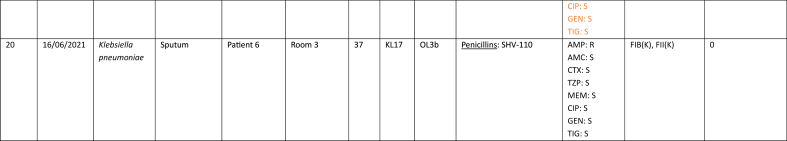
Epidemiological, genomic and phenotypic profiles of Klebsiella isolates recovered from patients and hospital sinks

R: resistant; S: susceptible; AMP: ampicillin; AMC: amoxicillin-clavulanic acid; CTX: cefotaxime; TZP: piperacillin-tazobactam; MEM: meropenem; CIP: ciprofloxacin; GEN: gentamicin; TIG: tigecycline.

**Figure 2:**
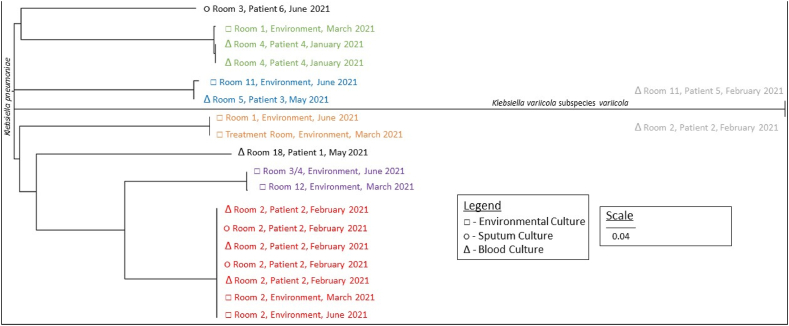
**Phylogenetic tree** Colours correspond to those used in Figure 1.

Colours correspond to those used in Figures 1 and 2.

Of the six patients included, three (patient 2, patient 4 and patient 5) were primarily admitted with COVID-19 pneumonitis necessitating intensive care, but in all cases, the Klebsiella-associated infection was a contributing factor to mortality. Infection with the identified Klebsiella strain was determined based on clinical judgement from the treating intensivist, respiratory physician and clinical microbiologist, to have caused or significantly contributed to this outcome.

WGS and SNP analysis revealed genetic relatedness between clinical and environmental isolates (Figure 2). Relatedness was inferred using established SNP thresholds for *K. pneumoniae*, with ≤10 SNPs denoting highly related isolates and ≤25 SNPs indicating close relatedness [37,38].

*K. pneumoniae* cultured from blood and sputum from patient 2 was found to be highly genetically related to *K. pneumoniae* cultured from the handwashing sink in patient 2's environment, namely in the antechamber of the lobbied single room of room 2. These isolates differed by 2–5 SNPs, consistent with recent transmission or a shared source. Furthermore, this environmental isolate persisted for at least 3 months, from March 2021 to June 2021 in the handwashing sink. These relationships are depicted in red in Figure 1, Figure 2.

A *K. pneumoniae* isolate cultured from the blood of patient 3 in room 5 was closely related to an environmental isolate recovered from the handwashing sink in room 11, differing by 23 SNPs (as depicted in blue in Figure 1, Figure 2). Similarly, a *K. pneumoniae* isolate obtained from the blood of patient 4 in room 4 was closely related to an environmental isolate from the handwashing sink in room 1, differing by 15 SNPs (as depicted in green in Figure 1, Figure 2).

Highly genetically related environmental isolates of *K. pneumoniae* were also present in handwashing sinks 3/4 and room 12, differing by 9 SNPs (as depicted in purple in Figure 1, Figure 2) and between handwashing sinks in room 1 and the treatment room, differing by 5 SNPs (as depicted in orange in Figure 1, Figure 2).

Notably, two isolates were identified as *K*. *variicola* subspecies *variicola* by WGS, which were initially identified as *K*. *pneumoniae* using the VITEK-MS MALDI-TOF. This misidentification is well recognised [39], and final identification was confirmed on repeat testing. These isolates of *K*. *variicola* subspecies *variicola* were cultured from blood from two spatially distinct patients – patient 5 in room 11 and patient 2 in room 2 – and were found to be highly genetically related, differing by only 2 SNPs. This relationship is depicted in grey in Figure 1, Figure 2.

Genomic features are summarised in Table I. Among the 20 isolates, eight distinct STs were observed. ST1695 (7/20) and ST23 (3/20) constituted the largest clusters. The most frequent capsule loci were KL163 (*N* = 7) and KL1 (*N* = 3), while O-antigen loci were predominantly OL2a.1 (*N* = 9) and OL2a.2 (*N* = 7). Plasmid replicons were common, most frequently FIB(K) (*N* = 13) and FII(K) (*N* = 12). The virulence-plasmid marker repB_KLEB was detected in three ST23 isolates, which also carried yersiniabactin, colibactin and aerobactin and/or salmochelin. Most other isolates lacked hypervirulence loci (*N* = 14) or carried yersiniabactin alone (*N* = 3).

Phenotypically, all isolates were resistant only to ampicillin, except for two ciprofloxacin-resistant ST307 isolates that harboured quinolone resistance-determining region mutations and plasmid-mediated fluoroquinolone resistance genes.

Importantly, clinical and environmental isolates shared highly similar genomic features within room-linked clusters, consistent with persistence in sinks and episodic transmission, as illustrated by the corresponding colour coding between Table I and Figure 2.

### Local consequence

Given the evidence of non-MDRO cross-transmission and implications for patient safety, IPC policies were reviewed, focusing on decontamination and cleaning practices. Previously, handwashing sinks in rooms without known MDRO-colonised patients were surface-cleaned daily with TASKI Sani 4 in 1 (Diversey) [40] and weekly with Actichlor (Ecolab) [41]. Those in rooms with MDRO-colonised patients were cleaned daily with Actichlor, including plumbing treatment. Following dissemination of our findings, daily Actichlor treatment of both sinks and plumbing in all augmented care areas [42] was implemented from July 2021. In parallel, targeted training sessions were delivered to clinical staff to increase awareness of the risks associated with water/wastewater reservoirs and to reinforce appropriate use of clinical handwashing sinks.

A brief follow-up investigation in November 2021 involved swabbing all ICU handwashing sinks to evaluate microbial burden after this policy change. *K. pneumoniae* was again isolated from sinks in the antechamber of room 2 and the treatment room. However, no clinical specimens from ICU patients yielded *Klebsiella* spp. during this period, suggesting an absence of ongoing cross-transmission but persistence of environmental colonisation within the plumbing system.

## Discussion

WGS in our study demonstrated clear genetic relatedness between Klebsiella isolates recovered from patients and handwashing sinks, indicating cross-transmission between clinical and environmental sources. This finding aligns with multiple outbreak investigations implicating hospital water/wastewater systems as reservoirs and vehicles for microbial transmission [11]. Understanding the mechanisms underlying such transmission is critical for informing effective IPC strategies.

The aqueous environment presents unique challenges to IPC, with wet surfaces providing the solid–liquid interface that predisposes to biofilm formation [4,43]. These biofilms, which can harbour both antimicrobial-resistant and susceptible organisms, may disperse through several mechanisms. The most common is splashing during routine sink use, but spread can also occur through poor practices such as rinsing medical equipment, filling patient washbowls or leaving items on sink ledges, where they can become contaminated by splashed water and act as secondary reservoirs for transmission. While airborne aerosolisation has been reported, it is considered a less likely route than splashing and direct contact. The design of sinks and drains is also an important factor. Shallow basins or drains positioned directly beneath taps increase the risk of splashing, whereas rear-drain sinks with impaired drainage create conditions for wastewater reflux and dispersal of organisms. In contrast, deeper basins with off-set rear drains and free-flowing plumbing may reduce but not fully eliminate these risks.

Water-related HCAIs comprise an estimated 22% of Centers for Disease Control and Prevention (CDC) HCAI outbreak investigations [44]. Extrapolated to European ICU settings, this corresponds to 8000 preventable deaths annually [45].

A narrative review encompassing 30 outbreak and intervention studies between 1998 and 2020 highlighted the role of handwashing sinks and associated pipework in facilitating pathogen persistence and dispersal [10]. In one outbreak from a Spanish ICU, 42 patients were affected due to contamination of water/wastewater pipework [46]. Another study involving 338 ICU patients in the USA found that 28% became colonised with MDROs traced to water/wastewater systems, which was significantly associated with increased 90-day mortality [47]. A more recent review provided an updated synthesis of a further 19 drain-related outbreaks reported between 2019 and 2024 [11]. The majority occurred in ICU settings and were similarly linked to MDROs, as well as additional cases involving *Serratia marcescens*, *Stenotrophomonas maltophilia* and *Acinetobacter baumannii*. In 17 of the 19 outbreaks, sink drains and associated fixtures were implicated, with environmental contamination also found in showers, toilet water and nearby equipment.

Notably, both reviews emphasised that outbreak control required combined, multimodal interventions. These included standard IPC measures, such as hand hygiene, PPE use and patient isolation, alongside engineering controls like sink removal, drain disinfection, physical barriers and sink redesign. Human behaviour also played a critical role. A review of handwashing sinks activities in the USA found that handwashing accounted for only 4% of all sink activities and that sinks were being used as disposal sites for residual medications, along with material from parenteral feeding and patient waste, further reinforcing the need for staff education [48].

In our ICU, patients are routinely washed at the bedside using single-use portable washbasins that are filled from handwashing sinks. The sinks are rear-drain, free-flowing and not located beside preparation surfaces. During audits, basins were filled appropriately without contact with drains and no inappropriate use of sinks for equipment rinsing or product disposal was observed. Nevertheless, practices may differ outside observation periods as behaviour is known to change under observation [49]. Several ICU outbreaks have implicated patient washbasins as vehicles for Gram-negative transmission, making this a plausible pathway [50,51]. Staff training on wastewater risks and appropriate sink use was therefore reinforced during the outbreak response.

To date, much attention has centred on MDROs, owing to their considerable clinical implications. However, non-MDROs also play a significant role in transmission and are generally more prevalent across healthcare settings [52]. The insensitivity of current surveillance systems for antimicrobial-susceptible organisms is illustrated by WGS investigations across hospitals within the UK, which revealed extensive transmission of antimicrobial-susceptible *Pseudomonas aeruginosa* from water outlets to patients. Genetically related strains were identified in three of four centres, yet local teams regarded these as sporadic cases, underscoring the limitations of conventional surveillance. Importantly, the affected patients developed bloodstream and respiratory tract infections, demonstrating that antimicrobial-susceptible organisms can cause significant clinical disease even when not captured by MDRO-focused surveillance [53]. These findings illustrate why non-MDR organisms, despite being historically overlooked, offer important opportunities to better understand transmission pathways and to inform more robust IPC strategies.

Furthermore, persistence of non-MDROs within sink and plumbing biofilms creates opportunities for horizontal gene transfer and acquisition of mobile genetic elements, with the potential for later emergence of multidrug resistance. The physicochemical conditions of wastewater biofilms, including high cell density, close proximity of diverse Gram-negative species and intermittent exposure to disinfectants and low-level antimicrobial residues, favour conjugation, integron activity and plasmid maintenance, so resistance determinants can spread even in the absence of therapeutic antimicrobial pressure [54].

Although direct causality is often difficult to confirm, several studies have shown reductions in HCAI rates following the removal of sinks and implementation of water-free care protocols. One quasi-experimental study reported a decrease in ICU-acquired Gram-negative bacilli from 26.3 to 21.6 per 1000 patient-days, with the most notable impact in patients with stays of ≥10 days, showing a 2.5-fold reduction [55]. Systematic and narrative reviews echo these findings, emphasising that water-free interventions are effective and necessary strategies to minimise waterborne HCAIs and improve patient safety [45,56]. Despite this evidence, most critical care units have introduced water-free care only reactively in response to intractable MDRO outbreaks. If antimicrobial-susceptible organisms are transmitted through the same environmental routes, a proactive approach is needed. In the absence of recognised ongoing transmission, the perceived urgency to change practice is often low. Evidence from a large multi-centre retrospective analysis of 552 ICUs demonstrated that the presence of sinks in patient rooms was independently associated with higher rates of HCAIs overall, as well as increased *P*. *aeruginosa* infections, even outside outbreak settings [57]. These findings highlight the risks posed by clinical handwashing sinks and support the argument that water-free care should be considered not only as a crisis response but also as a preventative strategy.

Water-free care is not currently implemented locally. The ICU plumbing is relatively old, which makes the adoption of engineering interventions challenging without major infrastructural refurbishment. There has also been staff hesitancy to move towards water-free care, highlighting the need for engagement and support from service users before such a fundamental shift in practice can be achieved. Resource limitations and the absence of national guidance or consensus on water-free care have further influenced decision-making. The proactive adoption of water-free care may be appropriate in some settings, though this requires significant infrastructural support and strong clinical engagement.

Moving forward, sustainable strategies should also be considered where feasible and practical. The regular long-term use of biocides in drains is unlikely to represent a viable solution as effectiveness is often inconsistent, and there are concerns regarding the potential for tolerance, resistance and broader environmental impacts [58,59]. Further options include novel technologies such as thermally disinfecting waste traps or vibrational units, as well as redesigned sinks with deeper basins and off-set drains, although these require substantial investment [22,60].

Our study has several limitations. Environmental sampling was limited to handwashing sinks and focused exclusively on *Klebsiella* spp., potentially underestimating the broader extent of contamination and the presence of other clinically relevant organisms. Furthermore, environmental samples were not collected prior to patient admission. As a result, we cannot determine the directionality of transmission – whether the environment served as the source or was contaminated by colonised patients; but what we can report is evidence of cross-transmission between patients and the hospital environment. In addition, colonisation data were not available beyond routine CPE screening, which restricted our ability to assess asymptomatic carriage and its potential role in sustaining transmission. Finally, only one colony per positive environmental culture was selected for sequencing due to resource limitations, which may have underestimated strain heterogeneity within biofilms where polyclonal and polymicrobial populations are common.

Despite these limitations, the use of WGS and SNP analysis enabled the identification of core-genome differences with high resolution, which provide support for environmental involvement in the transmission of GNOs. These findings highlight the need for expanded environmental surveillance where relevant, particularly in situations where sources of outbreaks are challenging to elucidate or during unusual clusters of unexpected clinical infections. They also underscore the importance of incorporating antimicrobial-susceptible organisms into IPC and healthcare epidemiology research. Focusing solely on MDROs may miss critical opportunities to understand and interrupt transmission pathways.

In conclusion, our study supports the need for a clearly defined IPC strategy to control environmental colonisation and onward transmission of GNOs. To date, there is a scarcity of randomised controlled trials examining the role of interventions targeting the environment, specifically in reducing rates of HCAIs, and more research is needed to quantify the proportion of transmissions via environmental contamination.

Most outbreak reports focus on MDROs, but our study highlights that non-MDROs should not be overlooked as they too persist and cause infection. It is likely that the role of the environment in propagating HCAIs is skewed toward MDROs, which most likely underestimates the true burden of all HCAIs, especially where non-MDR isolates were involved.

## CRediT authorship contribution statement

**Saied Ali:** Formal analysis, Resources, Writing – original draft, Writing – review & editing. **Guerrino Macori:** Data curation, Formal analysis, Methodology, Project administration, Resources, Software, Visualisation, Writing – review & editing. **Niamh Mullane:** Investigation, Methodology, Writing – review & editing. **Belsie Jayaseelan:** Methodology, Resources, Writing – review & editing. **Orla Donoghue:** Methodology, Resources, Supervision. **Seamus Fanning:** Conceptualisation, Funding acquisition, Project administration, Supervision, Validation, Writing – review & editing. **Kirsten Schaffer:** Conceptualisation, Investigation, Methodology, Project administration, Supervision, Writing – original draft, Writing – review & editing.

## Ethics statement

This is a retrospective study of a pseudonymised data set with neither human participants nor animals, and as such, formal ethical approval was waived by the local research ethics committee – St. Vincent's University Hospital Ethics Committee.

## Funding sources

This research did not receive any specific grant from funding agencies in the public, commercial, or not-for-profit sectors.

## Conflict of interest statement

None declared.
